# Hypersensitivity Reactions to Food Additives—Preservatives, Antioxidants, Flavor Enhancers

**DOI:** 10.3390/ijerph191811493

**Published:** 2022-09-13

**Authors:** Mateusz Witkowski, Halina Grajeta, Krzysztof Gomułka

**Affiliations:** 1 Department of Dietetics and Food Science, Wroclaw Medical University, 50-556 Wroclaw, Poland; 2 Department of Internal Disease, Pneumology and Allergology, Wroclaw Medical University, 50-369 Wroclaw, Poland

**Keywords:** allergy, hypersensitivity, food additives functions, consumer safety, therapy

## Abstract

There have been reports of food hypersensitivity reactions to food additives (HFA) for many years. The mechanisms of HFA and their frequency are difficult to precisely define, as most of the data come from outdated studies with poor methodology. In 2020, the European Food Safety Authority completed a review of additives, examining their influence on the occurrence of HFA, but did not include all of them. The aim of this review is to systematise knowledge about selected groups of food additives (FAs) and the HFA induced by them. We also briefly discuss the issues of diagnosis and therapy in this disease. FAs are commonly used in prosscessed foods, but HFA appears to be a rare phenomenon. Identification of the FA responsible for hypersensitivity and its treatment is difficult. Diagnosis is a challenge for the clinician and for the patient. A food diary is a helpful diagnostic tool. It allows diet therapy to be monitored based on the partial or complete elimination of products containing a harmful additive. An elimination diet must not be deficient, and symptomatic pharmacotherapy may be necessary if its application is ineffective. Taking all this into account, we conclude that it is necessary to conduct randomised multicentre studies based on the double-blind placebo control protocol in this field.

## 1. Introduction

With the development of the food industry, food additives have become ingredients of processed foods. For decades, food hypersensitivity reactions incident to the consumption of food products containing these substances have been observed. Unfortunately, the data on hypersensitivity related to the consumption of additives often come from a few studies involving small groups within the population; some of them are even casuistic papers, and they are mostly from the last decades of the previous century [1,2]. The results presented by other researchers are inconclusive and do not answer the question of whether a relevant additive may induce hypersensitivity reactions; therefore, this issue remains open. Simultaneously, it is not always known whether food additives cause hypersensitivity or exacerbate the symptoms of an existing disease, or whether the coexistence of several variables is necessary for adverse effects to occur.

Even though over a decade has passed since the publication of the classification of adverse food reactions by NIAID, the terms food allergy and hypersensitivity [3] are still used alternately, even in the professional literature, which is not always reasonable regarding the pathomechanism of a relevant disease. To a high degree, this is caused by the fact that the problem of additives in the context of how they induce unwanted symptoms is complex. It seems that hypersensitivity to food additives (HFA) is mainly related to the advancement of atopic skin diseases [2]. There are also known cases of contact dermatitis (type IV allergic reactions) after occupational exposure to additives [4,5,6,7,8], or the use of cosmetics in which the same additives may be present as in food [9,10,11]. Therefore, it should be noted that some food additives or their metabolites cause allergic reactions and others cause non-allergic ones. However, this mechanism is often not fully understood. In addition, although there is no evidence for this, many additives have been reported to lead to symptoms of food hypersensitivity [12,13].

At the end of 2020, a decade-long review of the safety of food additives by the European Food Safety Authority (EFSA) was completed. The review considered the effect of additives in the development of food hypersensitivity [14]. Unfortunately, contrary to the initial assumptions, so far it has not been possible to carry out appropriate analyses for some substances, including sweeteners. Regarding all these issues related to the problem of HFA, the aim of this paper is to systematise knowledge about the mechanisms of these reactions, while briefly reviewing individual groups of food additives and presenting additives that are responsible for individual clinical symptoms, taking into account the data from EFSA publications and other data from the literature. Required data about the effect of individual food additives on the occurrence of hypersensitivity reactions were searched/collected from the EFSA scientific opinions online database. Keywords used in the search were the names of preservatives, antioxidants and flavour enhancers used in the EU, or their E numbers. Moreover, such data were searched/collected from online databases including Wiley, PubMed and Google Scholar. In these cases, the same keywords were used as for the EFSA database search, but with the addition of words such as ‘allergy’, ‘hypersensitivity’, ‘intolerance’, ‘asthma’, ‘allergic reaction’, ‘angioedema’, ‘urticaria’ or ‘rhinitis’. Other keywords used in the research process were ‘pathomechanism of hypersensitivity to food additives’, or, e.g., ‘pathomechanism of hypersensitivity to sulphites’ and ‘epidemiology of hyper-sensitivity to food additives’. Some of the articles come from the collections of the Wroclaw Medical University Library or the authors’ own collections. All information was derived from selected original articles and reviews published in peer-reviewed journals from the past 30 years. Much of the data available online come from studies which are more than 10 years old, and a large portion are case studies, as can also be seen in the EFSA reports [15]. The remaining parts of the work will broach the issues of diagnostics and therapy in this group of diseases.

## 2. Epidemiology and Pathomechanism of HFA

Only some people are hypersensitive, and only to certain food additives—this means that not all foods containing food additives should be automatically excluded from the diet. For example, monosodium glutamate occurs naturally in sardines or parmesan cheese, and at a much higher concentration than when used as a food additive. Other ‘natural chemicals’ causing allergies or intolerances include nuts and seafood [12]. The frequency of reactions to food additives is estimated at less than 1% of the total number of food hypersensitivity reactions in adults and up to approximately 2% in children.

According to Kołodziejczyk et al. [16], HFA occurs in 0.5% of the general population and slightly more often in the atopic population. Moreover, Pałczyński suggests [17] that HFA is more common in some cohorts, e.g., in 25% of patients with a food allergy, 10–20% patients with IgE-dependent bronchial asthma, 5–10% patients with aspirin intolerance and 5–10% patients with chronic urticaria. According to other data, while the prevalence of food allergy in the general population is estimated at 5% [18], HFA is about 0.026% [19]. The lack of knowledge of their exact number is related to the difficulty in recognising them—it should be noted that hypersensitivity to food (also confirmed by a provocation test) is much less common than is shown in medical histories, and atopy is not a known risk factor for HFA [20].

Exposure to food additives is attributed to numerous symptoms, but the actual cause-and-effect relationship has not yet been well proven. Reactions to food additives should be suspected in patients who have been ruled out for a food allergy and who report symptoms after eating a variety of unrelated foods—especially when they concern store-bought food and do not include the same food prepared at home.

The pathomechanism of the reaction to food additives should allow for both the allergic background (with the dominant IgE-independent pathway) and the non-allergic background (toxic and immunotoxic—shown in Table 1) [21,22]. Symptoms caused or exacerbated by food additives are usually less dramatic than those caused by IgE-dependent allergic reactions, which also suggests mechanisms of non-IgE-dependent intolerance or allergy [23]. In a review study by Gostner at al. [24], it was shown that in vitro, not only typical antioxidants (e.g., vitamin C, E, resveratrol), but also some preservatives (i.e., sulphites, benzoates) and curcumin dye, enhance a suppressive effect on the activation of Th1 cells in freshly isolated human peripheral blood mononuclear cells, and additionally reduce the number of pathogens to which people are exposed. This process shows that antioxidants have an anti-inflammatory property, but on the other hand could shift the immune balance towards Th2-type immunity, which is an important pathway in allergic reactions. Because dietary habits and FA use have changed drastically, an increased tendency towards allergy and asthma in the Western world might be observed. The occurrence of symptoms, their nature and intensity are also influenced by so-called modulating factors, which include the amount and form of the consumed allergen, frequency of its consumption, cumulative effect and additional trigger mechanisms (physical activity, consumption of coffee or alcohol, menstruation) [25].

## 3. The Role and Types of Food Additives

There are over 300 food additives allowed for use in EU countries. According to the definition provided by Regulation (EC) No. 1333/2008 of the European Parliament and of the Council of 16 December 2008 on food additives, food additives ‘shall mean any substance not normally consumed as a food in itself and not normally used as a characteristic ingredient of food, whether or not it has nutritive value, the intentional addition of which to food for a technological purpose in the manufacture, processing, preparation, treatment, packaging, transport or storage of such food results, or may be reasonably expected to result, in it or its by-products becoming directly or indirectly a component of such food’ [26].

Food additives enable a reduction in the cost of food production and increase its efficiency, at the same time improving the food’s microbiological safety and chemical stability and giving it the appropriate organoleptic features. Currently, the topic of the health impact of food additives is still controversial and many myths have arisen around it, supported by easy access to information of dubious origin [25].

In the EU countries, EFTA, GCC, South Africa, the U.K., Australia, New Zealand, Malaysia, Hong Kong, India and Israel, as well as in the USA, appropriate numerical markings preceded by the letter ‘E’ are used for individual categories of food additives, depending on their technological function, and mainly in products of European origin. The scheme below (Table 2) presents a short description of the groups of food additives based on the Regulation (EC) No. 1333/2008 of the European Parliament and of the Council of 16 December 2008 on food additives. It should be noted that, contrary to popular opinion, compounds with an E-number include not only fully synthetic food additives, but also those that are obtained from natural raw materials, or are synthetic but identical to natural ones [26].

## 4. Adverse Reactions to Food Additives

Symptoms of HFA may affect one or more organs. The reaction to an allergen occurs up to 2 hours after its ingestion (early reaction), up to 12 hours after exposure (delayed reaction) or more than 12 hours after contact with the allergen (late reaction). The symptoms of food hypersensitivity concern not only the gastrointestinal tract, but may also affect the skin, respiratory system, central nervous system and circulatory system [27]. The most common clinical manifestations of reactions to food additives are chronic urticaria and angioedema, and, less often, anaphylactic reaction, an exacerbation of symptoms of atopic dermatitis, bronchial asthma or allergic rhinitis. In sensitive individuals, oral or local exposure may also cause several non-specific symptoms, such as paroxysmal facial flushing, hypotension, abdominal pain, deregulation of bowel movements, itching of the skin and tachycardia [28,29].

### 4.1. Preservatives

#### 4.1.1. Benzoic Acid

Benzoic acid (E210) and its sodium, potassium and calcium salts (E211–213) are used as preservatives due to their antimicrobial activity. According to the Commission Regulation (EU) No. 1129/2011 of 11 November 2011 amending Annex II to Regulation (EC) No. 1333/2008 of the European Parliament and of the Council, which established a European Union list of food additives, benzoates are added to various groups of food products, e.g., low-percentage alcoholic beverages, non-alcoholic beers, and low-sugar jams [30]. Benzoates can also occur naturally in foods; their source can be fermented milk products. In addition, benzoic acid derivatives can be derived from cinnamic acid [31,32].

The topic of hypersensitivity to benzoates has been a disputable issue among researchers for decades. According to some data [33,34,35], these substances contribute to the exacerbation of hypersensitivity reactions to certain drugs and foods containing benzoates, manifested in the form of various types of skin lesions (atopic dermatitis—AD) [33] or asthma exacerbation [34,35]. Unfortunately, these data were obtained based on a provocation test without a double-blind placebo-control (DBPC) challenge [34], or using this experimental method but giving patients with AD a mixture of several food additives, which could have significantly influenced the results of the study [33,35]. There are also reports of exacerbation of subjective and objective symptoms in patients with chronic non-atopic rhinitis (CN-AR) because of an E211 challenge using DBPC [35]. We found in the literature a case study based on the DBPC protocol, in which the occurrence of chronic E211-induced pruritus was observed [36]. In studies based on the E211 DBPC challenge in patients with a history of urticaria with or without angioedema, exacerbation of the disease in the form of urticaria was observed [28].

Why are the results of studies on the induction of food hypersensitivity by benzoates contradictory? According to [37], most benzoates in the human diet are derived from natural products, not food additives. Perhaps it is the accumulation of both E210 derivatives that leads to the exacerbation of an existing skin or respiratory disease [36].

In our opinion, this might be connected with another type of hypersensitivity, e.g., salicylate intolerance. Chemically, salicylic acid (2-hydroxybenzoic acid) is a derivative of benzoic acid. This is probably why some older studies [38,39] observed a co-occurrence of hypersensitivity to salicylates and certain food additives, including benzoates, while other publications do not show such a relationship [40,41]. Therefore, the issue of the coexistence of certain hypersensitivities is still a matter of dispute and requires careful investigation. EFSA also believes that benzoate intolerances are associated with an already ongoing disease process [42].

#### 4.1.2. Parabens

In food products, p-hydroxybenzoic acid esters (PHBA) and their salts (called parabens) are also used as preservatives. This group includes four substances: ethyl and methyl-p-hydroxybenzoate and their sodium salts (E214, E215, E218 and E219). They are used, for example, in potato and starch snacks and breath-freshening microsweets, as well as decorations, layers and non-fruit fillings in confectionery [30]. Apart from the food industry, they are widely used in cosmetics and therefore are amongst the most common contact allergens [43].

Their bactericidal and fungicidal activity are based on a change in the permeability of biological membranes, which adversely affects transmembrane transport. E210 derivatives are effective against gram positive bacteria, but less effective against bacteria than against fungi [44,45]. PHBA esters and their salts, after contact with the skin and after ingestion, are absorbed and metabolised to PHBA and p-hydroxyhipuric acid [46,47].

Over the past two decades, the occurrence of symptoms after oral paraben provocation has been described, and this concerned a subjective exacerbation of CN-AR [35]. In one of the more recent studies [48] conducted in a group of 100 patients with chronic idiopathic urticaria, no skin reactions to orally administered parabens were observed.

EFSA’s scientific opinion from 2004 [49] does not address the topic of hypersensitivity to this group of compounds. There is no strong evidence in the literature to suggest that parabens induce symptoms other than contact or drug-induced hypersensitivity. However, the available data suggest that some patients with idiopathic urticaria or rhinitis may develop or worsen symptoms of their underlying disease due to consuming a certain number of parabens. This should, however, be confirmed in such patients by a DBPC trial and an elimination diet test.

#### 4.1.3. Sulphites

Sulphites (E220–228) are mainly used as preservatives in fruit and vegetable products [30].

Microorganisms are able to pick up thiol groups from sulphur compounds. In microbial cells, these groups react with genetic material and proteins, which explains the antiseptic effect of sulphites. Moreover, these substances prevent oxidation and browning reactions by inhibiting the Maillard reaction and polyphenol oxidase [50].

According to EU regulations, the amounts of sulphites added to products are in the range of 10–2000 mg/kg of the product converted to SO2. In addition, they are used as carriers in food enzymes. Moreover, two sulphur compounds act as dyes—caustic sulphite caramel (E150b) and sulphite ammonia caramel (E150d). They can be used with many products of plant and animal origin in accordance with the quantum satis (q.s.) principle [22]. The presence of these substances in the diet is ignored in studies relevant to sulphite hypersensitivity, due to insufficient information on their metabolism [51].

Sulphites are responsible for 5–10% of asthma exacerbation, including severe episodes, after eating foods containing them or their inhalation. According to literature data, bronchospasm associated with their consumption may occur in up to 8% of asthma patients taking steroids. It has also been reported that one symptom after a sulphite challenge may be rhinitis [52,53,54]. While sulphites also exacerbate asthma in children, sulphite-dependent asthma is less common in the paediatric population than in the adult population [1,55]. However, some of the cited provocation attempts were not blind [52,54]. Skin symptoms such as redness, urticaria or angioedema might be also manifestations of hyper-sensitivity to sulphur compounds, at least in some patients [56].

One of the symptoms suggesting an immunological basis for sulphite hypersensitivity is also a generalised reaction (anaphylaxis), which was noted in several smaller studies [57,58], including a case study [57]. In one, life-threatening anaphylaxis was reported in a patient with mastocytosis [58]. Other systemic symptoms may include hypotension, heart rate fluctuations and fever. Hypersensitivity reactions to sulphites are also associated with gastrointestinal complaints, especially abdominal pain and diarrhoea, as well as nausea, vomiting and dysphagia [58,59,60].

EFSA, in its Scientific Opinion on the re-evaluation of sulphites as food additives, also notes that sulphites can exacerbate allergic symptoms, especially in patients with asthma, as well as in those with other atopic problems. In addition, experts note the symptoms of various organs and suggest that some may be genetically determined [61].

Allergic reactions to sulphites are rare in the general population and seem to be more common in people with allergies [62]. People who regularly use cosmetics or medications containing sulphites may present skin symptoms around the hands and face [59]. Sulphites may cause breathing difficulties within minutes after eating food containing them, and asthmatics and patients with a hypersensitivity to salicylates are at increased risk of reacting to sulphites; other potential symptoms include pharyngeal oedema, urticaria and anaphylactic reaction [63]. Sulphite sensitivity is more common in women than in men. Reactions to sulphites can range from mild to severe and even fatal bronchospasm in patients with asthma. However, even in patients who are sulphite-sensitive, the consumption of foods that contain sulphites may not produce a response, as it depends on many factors. The mechanism by which inhaled sulphites trigger bronchospasm is not clear. It may be due to the formation of sulphur dioxide in the respiratory tract, which affects the mucosa of the respiratory tract and to some extent activates both the IgE mechanism and the cholinergic reflex, causing bronchospasm [59,62,63,64,65].

#### 4.1.4. Nitrites and Nitrates

Sodium (E250) and potassium (E249) nitrites as well as sodium (E251) and potassium (E252) nitrates are another group of preservatives used mainly in the meat (nitrite) and dairy industry and in fish processing (nitrates). Their acceptable amounts for use or residues in the final product are from 50 to 180 mg/kg and from 10 to 500 mg/kg, respectively [30]. The mechanism of their inhibitory activity against microorganisms is not yet known, but they are capable of preventing the growth of pathogenic anaerobic bacteria [66].

Symptoms of hypersensitivity to nitrogen salts mainly concern pruritus [67], although there are also reports of angioedema and anaphylaxis [68] and other skin problems [33]. Juhlin [39] observed exacerbation of urticaria and/or angioedema in 6% of patients who were challenged with E249 and E251, respectively, and in 12% of these patients, the response was inconclusive. Perhaps these numbers would have been higher as a result of exacerbation of the disease, but some of the study participants had no symptoms during the course of the study. However, the available data refer mostly to sodium nitrate and/or nitrite and come from studies with methodological errors similar to those observed with other food additives [33,67,68].

EFSA, in its report [69] on nitrates, points at one work [70] and another work [71] in which it was noted that in vitro, peripheral blood mononuclear cells of healthy people influenced by E251 do not produce pro-inflammatory cytokines. The authors of the reports do not comment on the results of these studies, nor do they address the topic of nitrite hypersensitivity. In our opinion, at least some patients with chronic urticaria may show rare hypersensitivity reactions to this group of additives.

#### 4.1.5. Acetic Acid

Acetic acid (E260) is used as an acidity regulator and a preservative. It is added, according to the q.s. principle, to mozzarella cheese, whey cheese, preserved vegetables and fruit, to some raw meat products and bread, and as a pH regulator to cereal-based processed foods and baby food [30].

There have been reports of hives, anaphylaxis and asthma attack induced by acetic acid in people who are hypersensitive to ethanol. The reasons for these reactions are unknown, but case studies over the last decades of the previous century confirmed hypersensitivity to ethyl alcohol in open challenge tests and positive reactions to acetic acid in skin tests. These studies were carried out on people reporting the above-mentioned symptoms after consuming alcoholic beverages and foods containing vinegar [72,73]. Similarly, a positive skin test for acetic acid was found in a woman with urticaria and angioedema; however, no hypersensitivity to vinegar was observed in this case [74]. Thus, in our opinion, acetic acid hypersensitivity might be another extremely rare type of adverse reaction, or the previous data are the result of methodological errors and this topic needs to be further investigated.

### 4.2. Antioxidants

Antioxidants include substances that accept hydrogen, or an electron derived from other chemical compounds. In the form of a radical, they maintain chemical stability, which distinguishes them from other radicals. According to the Regulation of the European Parliament and of the Council (EC) No. 1333/2008 of 16 December 2008 on food additives, they are substances that protect food products from spoiling due to oxidation and discolouration [30]. Many different additives with antioxidant properties are used in the food industry [14,75].

#### 4.2.1. Gallates

Propyl gallate (E310) is present in, i.e., spice mixes and fats used in instant meals, especially stock cubes and soups, as well as in confectionery and other fats. In addition, it is used as an antioxidant in the cosmetics and pharmaceutical industries [1,30].

There are data [76] showing the induction of symptoms of contact dermatitis and dermatitis and cheilitis after ingestion of octyl gallate, currently not authorised in the EU as a food additive. There is also a possibility of the coexistence of hypersensitivity to E310—both substances were present in baking mixes, and after contact with them, symptoms were observed in patients. In a review of 74 cases of positive responses to gallate tests over a 40-year period, no hypersensitivity to E310 was observed after oral exposure. The main symptoms were skin contact reactions [77].

It seems correct to assume that gallates are not first-line substances in the diagnosis of hypersensitivity, and that foods containing E310 should not be routinely excluded from the diet of people with a tendency for skin lesions but should be considered in refractory reactions.

#### 4.2.2. Butylhydroxyanisole and Butylhydroxytoluene

The same products as E310 also contain butylhydroxyanisole (BHA, E320), which, along with butylhydroxytoluene (BHT, E321), belongs to the group of synthetic phenolic antioxidants. The addition of E321 is permitted, i.e., in some fats and spices [30]. In studies that investigated hypersensitivity to BHA and BHT, it was observed that these substances are responsible for exacerbating urticaria in some people. In one of the studies, the health condition of patients improved after dieting, with the exclusion of E320 and E321 [78]. An important discovery involving the use of the rat-derived RBL2H3 cell line was made by Yamaki et al. [79]. The results of this study indicate that BHT may contribute to the exacerbation of cutaneous anaphylaxis and the development of asthma and allergic rhinitis. Similarly, the occurrence of acute rhinitis was observed in a patient who was administered BHA orally in the DBPC test [54].

The EFSA issued opinions on E310 as well as BHA and BHT in 2011–2014 [80,81,82]. According to these documents, E310, based on the available literature, does not appear to be harmful to humans due to its tendency to induce symptoms of intolerance. Limitations in the availability of data on such activity for E320 and E321 mean that no conclusions can be drawn about any form of hypersensitivity to these substances after oral administration. In the opinion of the authors of this study—as suggested in the case of propyl gallate—the possibility of hypersensitivity should be considered in refractory cases, especially in cases where other causative factors have been excluded.

#### 4.2.3. Lecithins

Lecithins (E322) are used as antioxidants in fats, but they also have other technological functions. They are also used as emulsifiers or anti-caking agents. The main source of lecithin is soybean oil. On a smaller scale, lecithins are obtained from sunflower oil, rapeseed oil and eggs [83,84]. In other products, such as powdered milk, pasta, some breads, cocoa and chocolate products, the addition of lecithin is used according to the q.s. principle [30].

Soy protein residues may be present in soy lecithins. However, the addition of lecithins to food products is usually so small that it does not lead to hypersensitivity reactions even in people allergic to soy proteins. Nevertheless, particularly susceptible people may experience hypersensitivity reactions—including allergic ones—related to the consumption of food products containing E322 in their composition [85]. Consumption of soy lecithin may cause gastrointestinal symptoms of food allergy [84]. People who work in bakeries may experience asthma attacks from soybean lecithin [86]. One hundred milligrams of soy lecithin caused a rash on the face of a child with AD and asthma and peanut allergy undergoing DBPC. The rash also appeared on the body of another child with AD after DBPC with 50 mg of egg lecithin [87,88].

In the past decade, the EFSA has issued two documents related to the safety of lecithins [84,88]. They show that both egg- and soy-derived lecithins can induce allergic reactions. The Panel on Nutrition, Novel Food and Food Allergens deems it justified to minimise the residual allergenic proteins in lecithins used as food additives.

In clinical practice, when dealing with a patient with an allergy to egg or soy proteins, one should consider the possibility of hypersensitivity to products containing lecithin. Hence, diagnosis of such a patient should be considered to confirm the coexistence of this hypersensitivity.

### 4.3. Flavours Enhancers

#### Glutamates

Glutamates (E620–E625) are substances which generally do not have a strong taste but have the ability to intensify the taste of products and dishes. They are naturally found in seaweed, sauces of East Asian origin (fish, soy), some ripening cheeses, tomatoes, corn, green peas, green beans and scallops [89]. EU regulations allow the use of up to 10 g of glutamates/kg of product. The above restrictions do not apply to salt substitutes, spices and their mixtures or instant meals, in which the addition of glutamates can be used in accordance with the q.s. principle [30].

Monosodium glutamate (MSG) is responsible for the symptoms known as Chinese Restaurant Syndrome (CRS). This refers to a group of ailments that patients experience after eating foods rich in this chemical compound. These symptoms include headache and reddening of the skin on the face, hot flushes, a feeling of numbness in the neck radiating to the back and arms, numbness in the hands, tightness in the chest and even fainting [1,2]. The severity of symptoms depends on the amount of MSG consumed, so the amount in foods may not be enough to cause symptoms. For this reason, some experts dispute the existence of this disease [90,91]. On the other hand, the EFSA notes that there may be a percentage of healthy individuals or asthmatics with unstable disease who will be hypersensitive to oral doses of MSG >3000 mg, but the mechanism of inducing symptoms of this syndrome is unclear so far [92]. Similarly, some studies have observed an exacerbation or induction of skin symptoms in individuals with urticaria [54], while others have not confirmed such associations [93,94]. Data from the publication also indicate MSG as a causative factor of angioedema and allergic rhinitis [95]. It should be noted that some of these studies have some methodological limitations, e.g., the size of the studied population and the form of provocation [54,94,95,96].

Similar conclusions were drawn by EFSA experts assessing the safety of using glutamates as food additives. Although synthetic glutamate is chemically the same as that found naturally in food, it has yet to be conclusively confirmed that CRS is associated not only with synthetic MSG but also with its natural sources [92].

## 5. Diagnosis and Treatment

Due to the increasing number of substances added to food, as well as many possible pathomechanisms of their action, HFA is hard to diagnose and it is hard to statistically estimate its incidence. The basic role here is a detailed medical interview aiming to establish a cause and effect relationship between the onset of symptoms and the meal consumed.

A food diary may be a helpful tool in determining the cause of HFA symptoms and in further work with a patient suspected of such hypersensitivity. In this diary, the patient should write down what food products and dishes they consumed during the day, as well as their weight and volume expressed in home measures (e.g., glass, handful, plate, tablespoon). In addition, if possible, the patient should also write down the recipes of the consumed dishes and paste labels with the ingredients of the purchased products to it, in order to determine more precisely what may be the cause of their ailments. Any symptoms observed should also be recorded in this diary.

Skin-prick tests and the level of serum-specific IgE-antibody concentrations are commonly used in the diagnosis of food allergy, but they do not take into account preservatives added to food. In patients suspected with hypersensitivity to these preservatives, the ‘diagnostic gold standard’ is an oral challenge test with a DBPC trial—this test should be performed in a hospital ward [97,98].

Food additives are used in the amounts necessary for technological reasons. Food hypersensitivity induced by food additives is not a frequent clinical phenomenon, and its origin is unclear. An important issue related to HFA is the limited number of studies involving hypersensitive people that would enable clarification of the frequency of this type of hypersensitivity. However, if the patient’s hypersensitivity is confirmed, it is reasonable to follow a diet that eliminates additives that contribute to the occurrence of certain symptoms. Sometimes, this will mean the complete elimination of the additive, while sometimes it may be sufficient to limit it to a certain amount that is tolerated by the patient. However, such a diet cannot turn out to be deficient; hence, the care of a dietitian specialising in the diet therapy of this type of diseases is necessary. If such a procedure does not bring the expected results, the patient is at risk of severe reactions, or if the damages resulting from its use outweigh the benefits, symptomatic pharmacotherapy should be introduced [20].

## 6. Conclusions

Symptoms of HFA may have many types of manifestation, and the disease itself might have an early, delayed or late reaction. The consumption of FAs is rarely the single cause of chronic symptoms such as skin eczema, urticaria, angioedema or itching of the skin, and only exacerbates their course. Some symptoms might be an effect of the accumulation of consumption of food additives and of the same or similarly structured chemical compounds naturally found in food. Perhaps symptoms might be also the effect of interactions with other substances such as alcohol. HFA appears to be a rare phenomenon, but the results of its appearance might be a nuisance and even dangerous for the patient. Nevertheless, the current state of knowledge on the subject presented is insufficient. Many studies on this issue come from several decades ago and are based on outdated methodology. In our opinion, it is worth considering conducting randomised multicentre studies based on the DBPC protocol with the participation of people with idiopathic hypersensitivity or suspected hypersensitivity to specific additives, as well as patients in whom the symptoms of hypersensitivity are induced by unrelated commercial food products, while no symptoms are observed after eating the same food prepared at home. It should be noted that this kind of study would be a challenge for professionals due to the potential difficulties in obtaining a sufficiently large number of respondents, and such studies require planning over a period of several years or even a decade. These studies would not only update the data on the frequency of hypersensitivity to additives, but possibly confirm or refute current theories about their pathomechanisms and contribute to the development of less invasive diagnostic methods of similar accuracy. Hopes for the effective assurance of food safety in EU countries are raised by the activities of EFSA, which include the reassessment of the safety of additional substances and resulting changes in the food law. It should be noted that EFSA also relies on many studies from the previous century due to the lack of more recent data on this topic. However, we note that a greater problem for public health than the possible allergenic effects of food additives is ensuring the toxicological and microbiological safety of food. Besides, institutional action alone may be insufficient if society does not consciously make the right food choices based on labelling data. Therefore, health education, broadly understood, which takes into account nutritional components, may be crucial. In the diagnosis of hypersensitivity to food additives, a detailed nutritional interview and a tool such as a food diary, as well as a diet eliminating the food additives suspected of inducing disease symptoms may be helpful.

## Figures and Tables

**Table 1:** Types and characteristics of hypersensitivity reactions Own elaboration based on [22].

Type of Reaction	Type of Antigen	Involved Antibody or Cytokine	Involved Cells or Receptors	Example of Reaction
Type I	Soluble	IgE	Mast cells	Anaphylaxis, allergic rhinitis
Type II	Matrix- or cell-associated	IgG	Fc receptor and NK cells, phagocytes	Thrombocytopenia
Type III	Soluble	IgG	Fc receptor and complement cells	Arthus reaction
Type IVa	Direct T-cell stimulation or antigen presented by cells	IFN-γ, TNF-α	Macrophages	Contact dermatitis
Type IVb	Direct T-cell stimulation or antigen presented by cells	Il-5, Il-4 or Il-13	Eosinophils	Persistent asthma
Type IVc	Direct T-cell stimulation or antigen associated with cell	Perforin or granzyme B	T-cells	Contact dermatitis
Type IVd	Direct T-cell stimulation or soluble antigen presented by cells	GM-CS, CXCL8	Neutrophils	Stevens–Johnson syndrome

**Table 2:** Groups and technological functions of food additives. Own elaboration based on [26].

Group of Additives	Functions
Food dyes	They give a new or more attractive colour to food products.
	There are natural (including those identical to natural) and synthetic dyes.
	The dyeing ability and the durability of the colour of natural dyes depend on environmental factors. Synthetic dyes (organic and inorganic) allow obtaining a permanent colour, are cheaper and have a standard dyeing strength.
Preservatives	They prevent the occurrence of physical, chemical and biological changes in food, which affect the attractiveness and shelf life of food, thus extending it.
	They affect the structure and functions of microorganism cells and show mutagenic properties in relation to them. Their effectiveness is influenced by environmental conditions. Their activity depends on the type and strain of microorganisms. They are effective at concentrations < 2‰. Some of them give a sour taste to products.
Antioxidants, acids and acidity regulators	Antioxidants counteract the disadvantageous oxidation reactions of chemical compounds present in food products.
	Antioxidants include common antioxidants, substances with antioxidant properties in addition to other properties, and synergists.
	They must demonstrate the ability to maintain durability during technological processes.
	Acidity regulators and acids influence the acidity of food products.
Thickening agents, emulsifiers, emulsifying salts, leavening agent, moisture retainers and gelling agents	Thickening agents—allow obtaining the appropriate adhesiveness of food products.
	Emulsifiers—participation in the formation and/or maintenance of emulsions. Emulsifying salts—allow the homogeneous distribution of fats in cheeses through the dispersion of proteins. Some of them (as well as some preservatives and acidity regulators) increase the volume of products by releasing gases—the so-called leavening substances. Some keep the product moist, which prevents it from drying out. Gelling substances—giving food products in the form of a gel an appropriate consistency.
Anti-caking agents	They prevent food particles from sticking together.
Flavour enhancers	They intensify the flavour and the scent.
Sweeteners, glazing agents and others	The sweetness of sweeteners is close to or many times greater than that of sucrose. They are divided into natural, synthetic and semi-synthetic; the latter two types are used as food additives. Glazing agents allow the product to shine and protect its outer layer. Others include substances that contribute to the formation or prevention/reduction of foam formation.
Stabilisers and other additives	They allow maintaining the physical and chemical properties of food products. Carriers—enable the use of other substances by physically modifying food additives. Modified starches—starches subject to chemical modification, mainly used as thickeners. Packing and carrying gases—protect food against spoilage or enable stuffing products out of the packaging. Sequestrants—create chelates with metal ions, thus improving the oxidative stability of food and its quality. Improvers—improve the baking value of flour. Binding substances—help maintain the proper firmness of vegetables and fruit, facilitate the formation of gel synergistic effect for gelling substances. Bulking agents—increase the volume of the product.

## Data Availability

Not applicable.
